# One Tap at a Time: Correlating Sensorimotor Synchronization with Brain Signatures of Temporal Processing

**DOI:** 10.1093/texcom/tgaa036

**Published:** 2020-07-28

**Authors:** Gina M D’Andrea-Penna, John R Iversen, Andrea A Chiba, Alexander K Khalil, Victor H Minces

**Affiliations:** Neurosciences Graduate Program, UC San Diego, La Jolla, CA 92093, USA; Institute for Neural Computation, UC San Diego, La Jolla, CA 92093, USA; Neurosciences Graduate Program, UC San Diego, La Jolla, CA 92093, USA; Department of Cognitive Science, UC San Diego, La Jolla, CA 92093, USA; Institute for Neural Computation, UC San Diego, La Jolla, CA 92093, USA; School of Film, Music, and Theatre, University College Cork, Cork, T23 HF50, Ireland; Department of Cognitive Science, UC San Diego, La Jolla, CA 92093, USA

**Keywords:** electroencephalography (EEG), rhythm, sensorimotor synchronization (SMS), sensory integration, timing

## Abstract

The ability to integrate our perceptions across sensory modalities and across time, to execute and coordinate movements, and to adapt to a changing environment rests on temporal processing. Timing is essential for basic daily tasks, such as walking, social interaction, speech and language comprehension, and attention. Impaired temporal processing may contribute to various disorders, from attention-deficit hyperactivity disorder and schizophrenia to Parkinson’s disease and dementia. The foundational importance of timing ability has yet to be fully understood; and popular tasks used to investigate behavioral timing ability, such as sensorimotor synchronization (SMS), engage a variety of processes in addition to the neural processing of time. The present study utilizes SMS in conjunction with a separate passive listening task that manipulates temporal expectancy while recording electroencephalographic data. Participants display a larger N1-P2 evoked potential complex to unexpected beats relative to temporally predictable beats, a differential we call the timing response index (TRI). The TRI correlates with performance on the SMS task: better synchronizers show a larger brain response to unexpected beats. The TRI, derived from the perceptually driven N1-P2 complex, disentangles the perceptual and motor components inherent in SMS and thus may serve as a neural marker of a more general temporal processing.

## Introduction

Temporal structure provides a foundation for our experience of and interaction with the world. By developing temporal expectancies of stimuli in our environment, we prepare our attention and behavior appropriately (Jones and Boltz 1989). We can modulate our vigilance to co-occur with particular predictable events (Jones and Boltz 1989); we can coordinate our bodily movements over time and through space; we can parse and comprehend speech (Assaneo et al. 2019); and we can reorient to unexpected, potentially dangerous stimuli. Temporal processing exists through both conscious and early, low-level predictive processes and consequently offers a basis for perception and behavior. Furthermore, some have suggested that the brain’s ability to habituate to unimportant repeated stimuli and to orient to sudden changes (termed “neural adaptability”) reflects an energy-efficient strategy and allows fluid behavior in a changing environment (Schafer 1982; Záborszky et al. 2018). Understanding the neural basis of temporal processing thus may yield insight into human perception, action, and cognition.

Sensorimotor synchronization (SMS), the coordination of one’s movements with an external rhythm (Repp 2005; Repp and Su 2013), has been commonly employed to investigate timing ability in a number of experiments. Such studies, often exploiting the simplicity of finger tapping, reveal the role of temporal processing in a variety of abilities and pathologies: attention and attention-deficit hyperactivity disorder (Khalil et al. 2013), speech processing (Corriveau and Goswami 2009; Tierney et al. 2015; Zhao and Kuhl 2016), schizophrenia (Carroll et al. 2009; Papageorgiou et al. 2013), Parkinson’s disease (Roalf et al. 2018), and dementia (Rabinowitz and Lavner 2014), among others.

Because SMS involves both sensory and motor components, individual variation in synchronization may arise from differences in motor ability or from differences in the neural processing of time, and different paradigms are required to disambiguate the 2. A neural index of passive temporal processing could serve as a potential solution by disentangling temporal from motor execution. Electroencephalographic (EEG) experiments examining interval timing and rhythm suggest the N1-P2 complex to be a promising candidate due to its modulation by temporal expectancy (Schafer et al. 1981; Lange 2009; Todorovic and de Lange 2012; Kononowicz and van Rijn 2014; Duzcu 2019; Duzcu et al. 2019; Menceloglu et al. 2020). The N1-P2 complex, a brain evoked potential (EP) elicited by stimulus onsets, offsets, and change, reflects early sensory processing (Näätänen 1990; Remijn et al. 2014). This EP has been used to estimate auditory threshold in adults (Lightfoot 2016) and may be influenced by top-down attentional mechanisms (Hillyard et al. 1973; Parasuraman 1978). Moreover, the amplitude of the N1 may correspond to conscious perception, with larger amplitudes suggesting greater subjective obtrusiveness of a stimulus (Näätänen 1990). Accordingly, the present study examines the relationship between the N1-P2 complex and SMS, evaluating the potential of the N1-P2 to serve as a neural marker of timing ability and a window into the means by which temporal expectancy impacts the subjects’ ability to coordinate their actions with the dynamics of the environment.

The current experiment is thus designed to assess timing ability and to manipulate temporal expectancies. Participants first attempted to synchronize their tapping with an external beat on drums allowing digital recording. Subsequently, they partook in a passive listening task during which EEG was recorded: we adopted a temporal auditory oddball paradigm to measure neural responses to low-probability (“unexpected”) beats inserted within a stream of isochronous beats. In a previous study, Ford and Hillyard (1981) compared evoked responses to unexpected beats relative to expected beats in an isochronous stream. Although differences were observed, their study compared brain responses to beats with short and long interonset intervals (IOI’s); this approach conflates responses to broken temporal expectancies with the effect of sensory adaptation, a phenomenon in which responses to stimuli preceded by short IOI’s are smaller than responses to stimuli preceded by long IOI’s (Rothman et al. 1970). Our study intentionally evades differences in sensory adaptation to isolate the effect of temporal prediction on the N1-P2: We analyzed the neural response to a sound embedded in an identical local context in 2 conditions, one where it is unexpected and the other where it is expected, as determined by the global temporal context (see Methods). We hypothesized that better synchronizers would exhibit a larger N1-P2 complex to deviant beats.

## Materials and Methods

### Participants

A total of 35 participants between ages 18 and 24 (mean 19 years, 20 female and 15 male) were recruited from the University of California, San Diego, CA. One participant was excluded from the analysis due to excessive noise and artifacts in their EEG data, resulting in too few trials (<80) after artifact rejection. For efficiency and as part of a larger group dynamics experiment, all sessions were conducted in groups of 3, with the exception of 3 dyads. However, the tasks relevant to the present study entailed no explicit interactions between participants.

### Stimuli and Procedure

For both the tapping task and passive listening session, participants heard rhythmic streams of tone bursts from a speaker (~75 dB, sound pressure level). Each burst consisted of an 800-Hz sine pulse, 50 ms in duration with a 10-ms ramp up and ramp down to avoid clicks at onsets and offsets. All auditory stimuli and analog triggers were presented—and tapping recorded—using Adobe Audition with a Focusrite Scarlett 18i20 audio/digital interface with a sampling rate of 44.1 kHz. Stimulus presentation with a powered JBL studio monitor introduced a 3-ms latency. The auditory stimuli were sent to the speaker through one of the output channels of the audio digital interface. On another output channel, we sent a simultaneous analog trigger. The analog trigger was collected through one input channel of the audio digital interface, together with the activity from the piezoelectric elements that were glued to the tapping boards. With this configuration, the triggers and participants’ actions were collected simultaneously. Thus, the signals that we used for analysis were synchronized with a precision of one sample (i.e., less than a millisecond).

### Tapping

Participants first tapped individually for 1 min to isochronous beats. For the tapping task, beats were separated by a 600-ms IOI, a relatively comfortable pace for SMS (Repp 2005). Beats were occasionally omitted (15% probability); however, participants were instructed to maintain the basic rhythm while tapping and to tap where the beat “should be,” despite the omissions. Participants tapped onto wood blocks affixed to drums at approximately lap height, under which piezoelectric sensors were placed to record their taps as digital audio files into Adobe Audition.

### Passive Listening

**Figure 1:**
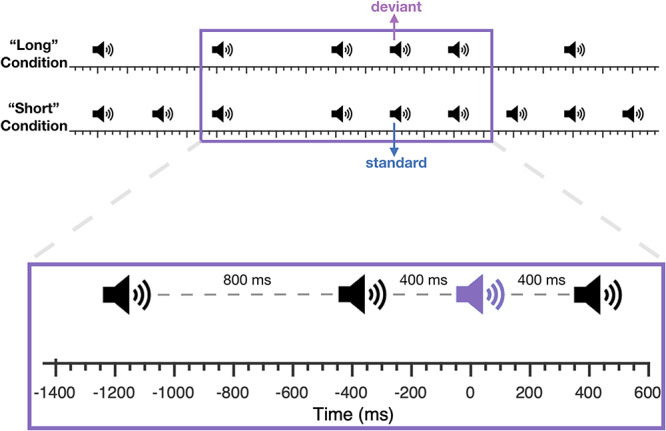
**Passive listening temporal oddball paradigm.** Participants passively listen to streams of sounds in 2 conditions. The “long” condition includes isochronous beats that are sometimes interrupted by an unexpected beat (“deviant”). The “short” condition is twice as fast as the long condition and contains occasional omissions. This arrangement allows us to compare brain responses with deviants and standards, which are physically the same sequence of stimuli to control for neural adaptation (see Methods) but vary in temporal expectancy.

A 30-min passive listening session followed the tapping task (Fig. 1). To avoid the confound of sensory adaptation, in which EP amplitude decreases with shorter IOI’s (Rothman et al. 1970), we developed a two-condition paradigm consisting of 2 trial types that were presented alternately 8 times (for a total of 16 trials). The “long condition” used a 800-ms IOI, with rare extra beats occurring at a 400-ms IOI (halfway between surrounding 800-ms IOI beats, 15% probability). The “short condition” used a 400-ms IOI, with rare omissions (15% probability). These 2 conditions strategically allowed for identical stimulus sequences in which the same 2 IOI’s preceded the key sound to which the evoked brain response was measured (either an extra beat in the long condition or a standard in the short condition), controlling for adaptation such that only their expectancy differed. Henceforth, the extra beats in the long condition will be referred to as “deviants” and the sequence-matched, expected beats in the short condition will be called “standards.”

### E‌EG Acquisition

**Figure 2:**
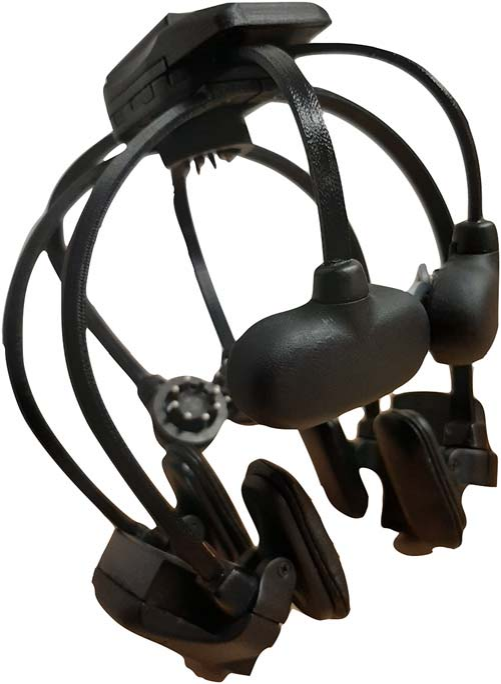
**EEG equipment.** Mobile, dry EEG headsets designed by Cognionics and employed to collect neural data.

During passive listening, participants wore custom, 5-channel dry EEG headsets containing electrodes at Fp1, Fp2, Cz, O1, and O2 (Cognionics; Chi et al. 2013; Fig. 2). Two frontal electrodes served as ground, and EEG data was referenced to the left earlobe. The headsets employ flexible plastic sensors with tines that penetrate hair to contact the scalp and active Ag/AgCl electrodes. Each headset was connected to a laptop using a USB isolator and running Cognionics Data Acquisition software. Cz was positioned approximately at vertex, and alcohol pads were used to clean participants’ foreheads and other electrode sites when necessary. Signal quality was inspected prior to recording: All impedances were below 400 kΩ, with most below 300 kΩ. Square-wave triggers, with variable amplitude serving as event codes, were sent into the headset as analog input and recorded as an additional channel. Data were recorded at 4000 Hz to preserve the onsets of the triggers.

## Analysis

### Tapping

An average of 122 taps was recorded per participant (range = 99–136). Tapping time series underwent a vector strength (VS) analysis, which provided a measure of each participant’s ability to synchronize with the external rhythm—particularly of their ability to tap with a consistent phase relative to the driving beat. VS is defined as:

$$ \mathbf{VS}=\frac{\mathbf{1}}{\boldsymbol{N}\ }\ \left|{\sum}_{\boldsymbol{j}=\mathbf{1}}^{\boldsymbol{N}}{\mathbf{e}}^{\boldsymbol{i}\boldsymbol{\phi } \boldsymbol{j}}\right|, $$

where *N* is the total number of taps by one participant, ϕ*j* is the tap phase relative to the driving rhythm for tap number *j*, and *i* is the imaginary unit. Accordingly, VS is one if a tapper is perfectly consistent, maintaining the same phase across taps, and zero if the participant taps randomly (Khalil et al. 2013). Circular standard deviation (SD) was also computed and yielded comparable results in the later correlational analyses.

### E‌EG

#### Preprocessing

EEG data was processed and analyzed using EEGLAB (Delorme and Makeig 2004). We used data from Cz, consistent with previous studies examining the N1-P2 complex (Hillyard et al. 1973; Parasuraman 1978; Schafer et al. 1981). After identifying the different types of events (standard and deviant beats) using the amplitude-coded square waves from the trigger channel, data were downsampled to 500 Hz. A low-pass Hamming windowed sinc finite impulse response (FIR) filter with a cutoff frequency of 30 Hz was applied to the data prior to event selection and epoching. The deviant and standard beats (Fig. 1) were selected, epoched, and baseline corrected to the 50-ms period preceding the beat. Initially there were 169 deviant events and 185 standard events for each participant. Automatic artifact rejection via extreme values removed trials with voltages beyond ∓100 μV, leaving a mean of 156 deviants (range 88–169) and 170 standards (range 81–185) per participant.

#### N1-P2 EP Identification

**Figure 3:**
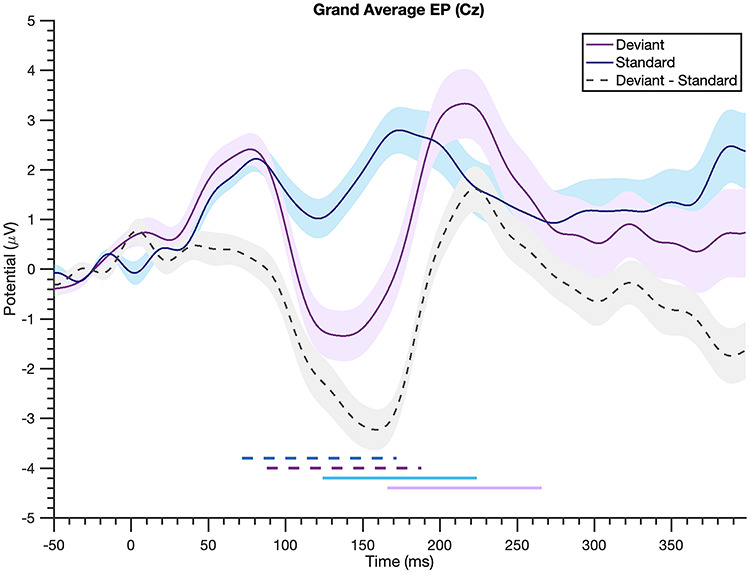
**Grand average evoked potential is larger to deviant beats.** Evoked potentials averaged across all participants (“deviant” = unexpected beat in “long” condition, “standard” = expected beat in “short” condition, shading = standard error). The N1-P2 complex (a negative-going deflection around 100-ms poststimulus and the following positivity) is noticeably larger to the deviant beats relative to the standards, as seen in the gray dashed difference wave. The horizontal lines at the bottom indicate the time windows used for mean amplitude measures: dashed dark blue for the standard N1, dashed purple for the deviant N1, solid light blue for standard P2, and solid light purple for deviant P2.

Across-participant grand averaged EPs were computed for the deviant and standard beats separately, and the resulting N1 and P2 for each event type were used to determine time windows for calculating mean amplitude measures in single participants’ EPs. Mean amplitude was used rather than peak values to bypass the influence of noise on single voltage values (Luck 2014); and separate time windows were examined for deviant and standard events, as N1-P2 latencies for the standard beat were generally earlier than those for the deviants (visible both in the grand average EP and in individual EPs; see Fig. 3 for grand averages). Time windows +/−50 ms surrounding the grand average peak for each condition were identified. Accordingly, the N1 mean amplitude for each participant was calculated between 72 and 172 ms and between 88 and 188 ms for the standard and deviant beats, respectively. The P2 mean amplitude was calculated between 124 and 224 ms for the standards and between 166 and 266 for the deviants. The N1-P2 magnitude was obtained by subtracting the N1 mean amplitude from the P2 mean amplitude, and a within-participant “difference magnitude” was calculated by subtracting the standard N1-P2 magnitude from the deviant N1-P2 magnitude. To evaluate the robustness of our findings using this difference magnitude measure, we both varied the window widths (+/−20 and +/−30 ms) and identified participants’ peaks. For the latter analyses, the N100 and P200 for each participant were determined as the minimum and maximum voltages between the time windows defined for mean amplitude, respectively. Difference magnitudes were then computed using these peaks as well as using the mean voltage 20 ms around these peaks.

### Statistics

The N1-P2 difference magnitude values between the deviant and short beats were correlated with solo tapping VS scores, using Spearman to account for non-normality and skew in VS scores. Supplementary analyses correlated the N100 and P200 magnitudes individually with VS and correlated the N1-P2 difference magnitude for each condition with VS. One-tailed *t*-tests comparing the N1-P2 magnitudes in the deviant and standard conditions and comparing the difference magnitudes of the 10 best and 10 worst synchronizers were conducted. We selected one-tailed *t*-tests because we hypothesized that deviant beats would elicit a larger N1-P2 and that this increase in magnitude would correspond to tapping ability, as better temporal prediction may underlie both measures. Although the *t*-test comparing the best and worst tappers is redundant following the correlational analyses, it accompanies a plot that allows for a better visualization of our findings.

Data and Code. Data and Matlab code for the analyses can be accessed at https://github.com/victorminces/TRI.

## Results

### N1-P2 to Deviants Versus Standards

To examine whether deviant beats elicit a larger N1-P2 than standard beats, we analyzed the N1-P2 magnitude to these types of stimuli. Consistent with dynamic attending theory (Jones and Boltz 1989) and the notion of neural adaptability (Schafer 1982), the N1-P2 magnitude to deviant beats (mean = 2.28, SD = 2.02) across subjects was larger than the N1-P2 to standard beats (mean = 0.36, SD = 1.19). This difference was statistically significant [paired one-tailed *t*-test, *P* = 1.22e-07, *t*-statistic = 6.47, df = 33], indicating that unexpected beats elicit a larger N1-P2 than their expected counterparts (Fig. 3).

### VS

VS was used to assess the ability to synchronize with an external rhythm. The mean and median values for VS were 0.82 and 0.89, respectively, with an SD of 0.187. VS scores were not normally distributed (*P* = 0.0017, Lilliefors test statistic = 0.198) and were left skewed (−1.5 skewness).

### Correlation Between N1-P2 Response and VS

**Figure 4:**
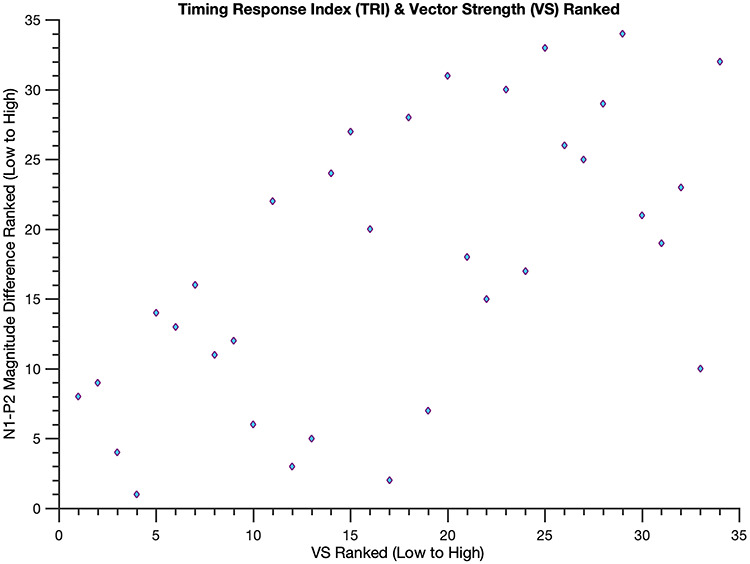
**Brain responses to unexpected beats predict better sensory motor synchronization.** Scatterplot of ranked VS and TRI values: lower VS values indicate poor synchronizing skills and lower TRI values indicate that the brain responses to unexpected beats are similar to expected beats. Better synchronizers (i.e. participants with higher VS scores) tend to exhibit a larger TRI (Spearman’s ρ = 0.61, i.e., a larger N1-P2 to deviant beats; *p* = 1.79e-04).

**Figure 5:**
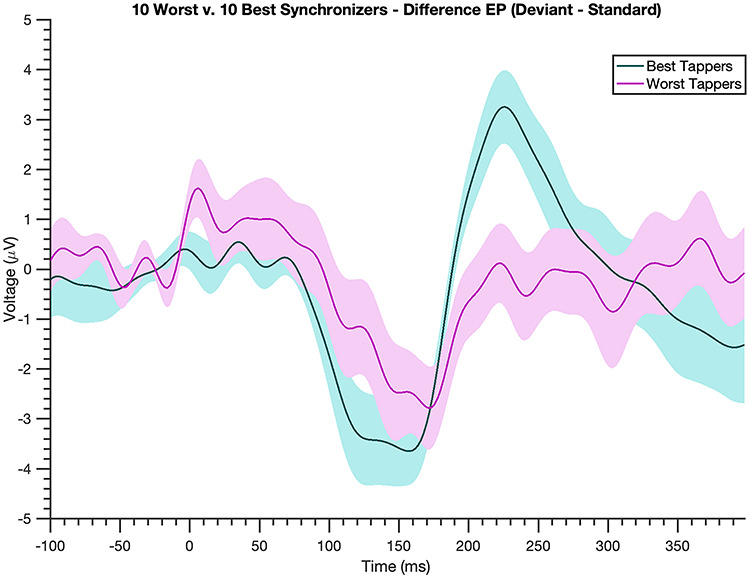
**Top synchronizers show a larger brain response to deviant beats.** Difference wave (EP to deviant beat–EP to standard beat, shading = standard error) for the best 10 synchronizers (teal) and worst 10 synchronizers (magenta) determined by VS scores. The best synchronizers exhibit a much larger N1-P2 complex to deviant beats compared with the worst synchronizers.

The N1-P2 difference magnitude significantly correlated with VS [Spearman’s ρ = 0.61, *P* = 1.79e-04]: Individuals with larger N1-P2 mean amplitudes to the deviant beat had higher VS scores (Fig. 4). That is, better synchronizers tended to have larger N1-P2 components to deviants. This correlation withstood a variety of manipulations, using shorter mean amplitude window widths [60-ms window, *P* = 0.02; 40-ms window, *P* = 9.74e-05], computing the N1-P2 difference magnitude with N1 and P2 peak voltages (*P* = 0.045) and averaging 20-ms windows surrounding individual participants’ peaks (*P* = 0.015), and including musical experience as a covariate [*P* = 8.21e-04]. Comparing N1-P2 difference magnitudes in participants with the 10 highest and 10 lowest VS scores yielded comparable results [one-tailed *t*-test, *P* = 1.25e-04, t-statistic = 4.55, df = 18]: Those with the highest VS scores had larger N1-P2 difference magnitudes than those with the lowest VS scores (Fig. 5), further illuminating the prior correlation. Upon examining the standard and deviant N1-P2 magnitudes separately, only the deviant N1-P2 magnitude significantly correlated with VS (ρ = 0.43, *P* = 0.013 for the deviant; ρ = −0.18, *P* = 0.30 for the standard), suggesting the effect is indeed driven by a larger N1-P2 to the deviant beat. Moreover, correlating the N1 magnitude and P2 magnitude individually with VS revealed that neither component alone is responsible for these findings (ρ = −0.11, *P* = 0.5 for the former and ρ = 0.3, *P* = 0.08 for the latter), an observation reinforced by a significant correlation between N1 and P2 magnitudes (*P* < 0.01 for both the deviant and standard, as well as for their difference).

## Discussion

Our results reveal a larger N1-P2 complex to deviant beats and find individual differences in this N1-P2 modulation to correlate with tapping ability measured as VS: Participants with a larger N1-P2 to deviant beats tapped more synchronously with an external beat. Overall, the larger N1-P2 complex to deviant beats is consistent with a variety of studies utilizing oddball paradigms (Ford and Hillyard 1981; Menceloglu et al. 2020), examining other temporal expectancy effects (Schafer et al. 1981; Lange 2009; Todorovic and de Lange 2012), and investigating interval timing (Kononowicz and van Rijn 2014; Duzcu 2019; Duzcu et al. 2019). We propose that our N1-P2 difference measure defines a timing response index (TRI).

Of greater interest is that the TRI predicts individual differences in tapping ability. The N1-P2, indicative of sensory processing, allows us to dissociate the sensory and motor aspects of temporal processing that are conflated in SMS. It is important to emphasize that the EP data analyzed was collected during a passive temporal auditory oddball session, separate from the active tapping task. Correspondingly, neural processing that occurs passively, reflecting the ability to detect temporal regularities in the environment, has significant implications for behavior. Maintaining SMS requires significant and continuous multisensory integration of information of different latencies and temporal resolution. The TRI, consequently, may serve as a neural marker of more generalized temporal processing, which may prove useful both theoretically and clinically, pending future empirical investigation.

It is possible that the TRI originates from other overlapping EEG components, such as the mismatch negativity (MMN), which may augment the N1. Yet, because the MMN has relatively poor signal-to-noise ratio and low intra/inter-subject reliability (Martin and Boothroyd 1999; Jones et al. 2000; Cone-Wesson and Wunderlich 2003), it is somewhat unlikely that this component accounts for our findings. Regardless, the predictive utility of the TRI endures whether it represents a genuine N1-P2 complex or some other component, and further research is required to assess its specific underlying brain potentials. Despite this qualification, our strategic passive listening paradigm circumvents the problem of sensory adaptation, in which shorter IOI’s in repeated stimuli elicit smaller neural responses such as the N1: we compare key beats (either labeled “deviants” or “standards”) that are preceded by the same sequence of stimuli and differ only in expectancy. The TRI thus cannot relate to differences in the physical stimuli themselves.

The TRI may signal the degree to which a given beat is detected as violating its temporal context. Such a violation requires additional processing, resulting in a cascade of incrementing attention, learning, and plasticity for the purpose of updating perceptual predictions. Greater sensory processing of, and attention to, less expected events may constitute an evolutionarily adaptive phenomenon requisite for learning, as such events may pose a threat or opportunity (Oros et al. 2014). In contrast, when innocuous stimuli, like the standard beats, are repeated, it would be neurally and behaviorally more efficient to reduce processing and perception of them unless they are task relevant. This supposition is also consistent with the musicological theory that intentional irregularity in music drives audience engagement and musical affect (Keil 1995). The coordination of temporal processing with perception, attention, and learning facilitates a more energetically economical and behaviorally efficacious means of interacting with our environment: we can either “tune in to” or “tune out” predictable stimuli while updating our expectancies to incidentally detected changes in the environment (Khouri and Nelken 2015).

Employing a tapping task and passive temporal auditory oddball paradigm, our study found that individuals with a larger N1-P2 complex to unexpected beats synchronized more adeptly with a driving rhythm. The N1-P2, associated with low-level sensory processing, may potentially signify the means by which temporal expectancy influences perception and contribute to our neural marker of temporal processing, the TRI. The rhythmic auditory task places no overt requirements on the subjects, requiring only passive listening, making the TRI particularly well suited for studying time processing in diverse populations (Kraus and Nicol 2017). For example, the TRI may facilitate the identification of temporal processing deficits clinically, both in communicative, compliant adults, and in noncompliant or vulnerable populations with compromised attention, cognition, behavior, and/or language (Carroll et al. 2009; Corriveau and Goswami 2009; Khalil et al. 2013; Papageorgiou et al. 2013; Rabinowitz and Lavner 2014; Roalf et al. 2018). Furthermore, examining the TRI in individuals trained to improve SMS may provide insight into the processes underlying such improvement. The reported correlation between the TRI and timing ability offers a variety of avenues to further explore the neural predictive processes crucial to motor coordination, dynamic attention, and cognition.

## Notes

We would like to thank Drs Tom Urbach, Marta Kutas, Virginia de Sa, Brad Voytek, and Laleh Quinn for their feedback and expert advice. In addition, we would like to thank the many interns who provided technical assistance: Britney Buu, Julia Chen, Nimisha Devanagondi, Holly (Yueying) Dong, Bahar Fouladpouri, Patricia Hsieh, Jorge Martin, and Angelo Salinda. *Conflict of Interest*: None declared.

## Funding

The National Science Foundation (NSF) (grant Social Behavioral and Economic Sciences Office of Multidisciplinary activities (SMA) 1041755 to the Temporal Dynamics of Learning Center for A.A.C., V.H.M., A.K.K., J.R.I.; SMA 1540943 for SL-CN: Group Brain Dynamics in Learning Network to J.R.I., A.K.K.); IBM Cognitive Horizons award to University of California, San Diego (UCSD) Artificial Intelligence for Healthy Living Center (to A.A.C.); associated San Diego Foundation fellowship award (to G.D.P.); Training Program in Cognitive Neuroscience National Institute of Mental Health (NIMH) (a Trainee Award T32 5T32MH020002-19 to G.D.P.).

## Notes

All contributed as senior authors
